# Cervical nodal volume for prognostication and risk stratification of patients with nasopharyngeal carcinoma, and implications on the TNM-staging system

**DOI:** 10.1038/s41598-017-10423-w

**Published:** 2017-09-04

**Authors:** Hui Yuan, Qi-Yong Ai, Dora Lai-Wan Kwong, Daniel Yee-Tak Fong, Ann D. King, Varut Vardhanabhuti, Victor Ho-Fun Lee, Pek-Lan Khong

**Affiliations:** 1 Department of Diagnostic Radiology, Li Ka Shing Faculty of Medicine, The University of Hong Kong, Hong Kong, Hong Kong SAR China; 2 Department of Imaging & Interventional Radiology, Faculty of Medicine, The Chinese University of Hong Kong, Hong Kong, Hong Kong SAR China; 3 Department of Clinical Oncology, Li Ka Shing Faculty of Medicine, The University of Hong Kong, Hong Kong, Hong Kong SAR China; 4 School of Nursing, Li Ka Shing Faculty of Medicine, The University of Hong Kong, Hong Kong, Hong Kong SAR China

## Abstract

We aim to evaluate the quantitative parameters of ^18^F-FDG PET/CT (metabolic parameters) and MRI (morphologic parameters) for prognostication and risk stratification in nasopharyngeal carcinoma (NPC). 200 (147 males, aged 50 ± 13 years-old, mean ± S.D.) newly diagnosed patients with NPC (T_x_N_x_M_0_) were prospectively recruited. Primary tumor and nodal lesions were identified and segmented for both morphologic (volume, VOL) and metabolic (SUV and MTV) quantification. Independent predictive factors for recurrence free survival (RFS) and overall survival (OS) were morphologic nodal volume (VOL_N, p < 0.001), TNM-stage (p = 0.022), N-Stage (p = 0.024) for RFS, and VOL_N (p = 0.014) for OS. Using Classification and Regression Tree (CART) analysis, three risk-layers were identified for RFS: Stage I/II with VOL_N < 18cc (HR = 1), stage III /IV with VOL_N < 18cc (HR = 2.93), VOL_N ≥ 18cc (HR = 7.84) regardless of disease stage (p < 0.001). For OS, two risk layers were identified: VOL_N < 18cc (HR = 1), VOL_N ≥ 18cc (HR = 4.23) (p = 0.001). The 18cc threshold for morphologic nodal volume was validated by an independent cohort (*n* = 105). Based on the above risk-classification, 35 patie*n*ts (17.5%) would have a higher risk than suggested by the TNM-staging system. Thus, morphologic nodal volume is an important factor in prognostication and risk stratification in NPC, and should be incorporated into the staging system, while PET parameters have no advantage for this purpose in our cohort.

## Introduction

Nasopharyngeal carcinoma (NPC) is prevalent in southern China, Taiwan and south-east Asia, with 80% of the world’s new cases from these regions^1^. Unlike other head and neck cancers, NPC is highly radiosensitive, and surgery is limited to salvage treatment for recurrent diseases^2^. The advancement in radiotherapy techniques and the use of concurrent chemotherapy has led to dramatically improved prognosis. However, outcome remains dismal for the 15~30% of patients who relapse after primary treatment^2^. With more aggressive treatment strategies and novel treatment options being made available, accurate prognostication and risk stratification are desirable.

The pivotal role of diagnostic imaging is in tumor staging, which is currently based on TNM staging system 7^th^ edition^3^. Due to its excellent soft tissue contrast and spatial resolution, MRI is the first choice for primary tumor delineation^4^. For nodal staging,^18^F-FDG PET/CT has been found to improve the diagnostic accuracy by providing metabolic information in addition to size criteria and morphology. In advanced disease, whole-body PET/CT offers an accurate means to determine distant metastases^5^. Apart from their respective roles in lesion detection and delineation, studies have found both modalities to offer useful quantitative parameters for treatment adaption and prognostication, e.g. standardized uptake value (SUV) on PET, and volumetric parameters on MRI and PET were found to be predictive of survival^6–8^. However, these studies investigated the imaging modalities independently, and to date, there has been no study that systematically compares the parameters from both MRI and PET/CT in NPC.

The limtations of the current TNM system for staging NPC is well-recognized and the need for modifications in the staging system have been suggested to better suit contemporary clinical practices which includes the incorporation of modern imaging in routine patient care^2, 9^. For example, the ability to measure discrete lymph nodes by imaging as opposed to clinical palpation predisposes N-staging to the lower N-stages compared to clinical staging^10^. Thus, a standard for imaging based staging, in particular for N-staging, is yet undefined.

We aim to explore the role of quantitative imaging parameters in a cohort of prospectively recruited newly diagnosed NPC patients for prognostication and risk stratification. In view of the limitations in the current staging system, we also explored if these modern imaging parameters could be used to improve the TNM-staging system.

## Material and Methods

### Patients

The study was approved by the Institutional Review Board of the University of Hong Kong/Hospital Authority Hong Kong West Cluster (HKU/HK HKW IRB), to include a primary prospective cohort and a retrospective cohort as a validation cohort, with written informed consent obtained for all prospectively recruited patients, and waived for the retrospective cohort. All procedures were in accordance with ICH GCP guidelines.

All consecutive adult patients (≥18 years of age) with treatment-naïve and histologically-confirmed NPC were recruited for the primary cohort. All patients in this cohort had both a standardized head & neck contrast-enhanced MRI and whole-body^18^F-FDG PET/CT within one week between scans for initial clinical staging and treatment planning from March 2008 to December 2014. Patients with distant metastasis (M1) or synchronous malignancies, subsequent non-completion of radiation therapy or lost to follow-up were excluded.

### Imaging protocols

Imaging acquisitions were performed using a 3-Tesla MRI (Achieva, Philips Healthcare, B.V., The Netherland) and 64-MDCT PET/CT (Discovery VCT, GE Healthcare Bio-Sciences, NJ, USA).

#### MRI protocol

Patients were scanned using a neurovascular coil (16-channel) with a scanning range covering the head & neck region from suprasellar region to apices of lungs. Imaging acquisition with standardized sequences was performed before and/or after intravenous bolus injection (0.1 mmol/kg at 1.5 ml/s) of DOTAREM^®^ (Guerbet, Villepinte, France). Details of the dedicated sequences are as follows:

- Pre- and post- contrast T1-weighted turbo spin echo (TSE) in axial plane, with repetition time/echo time (TR/TE) = 454/9.2 milliseconds (ms); turbo factor = 3; field of view(FOV) = 230 × 230 millimeter (mm); matrix = 672 × 672; slice number = 32; slice thickness = 3 mm; and intersection gap = 0.3 mm;
- T2-weighted short TI inversion recovery (STIR) in the axial plane, with TR/TE = 4644/60 ms; FOV = 230 × 230 mm; matrix = 672 × 672; slice number = 32; slice thickness = 3 mm; and intersection gap = 0.3 mm;
- T2-weighted STIR in the coronal plane: TR/TE = 4644/60 ms; with FOV = 230 × 230 mm; matrix = 480 × 480; slice number = 32; slice thickness = 3 mm; and intersection gap = 0.3 mm.

#### PET/CT protocol

^18^F-FDG was intravenously administered with a body-weight adjusted protocol (4.8MBq/Kg) after fasting for at least 6 hours. Serum blood glucose cut-off was 10 mmol/l, and the uptake-time was 1 hour. CT acquisitions (120 kVp, 200–400 mA, 0.5 s per CT rotation, pitch 0.984:1, 2.5mm intervals, with CT contrast medium injected at the dose of 1.5 ml/Kg) followed by PET acquisitions (2 min 30 s per bed position and 6 bed position per case) with coverage from base of skull to upper third of thighs. PET image acquisition was attenuated by CT using ordered-subset expectation maximization iterative reconstruction algorithm (14 subsets and two iterations). The resultant PET images were fused with CT images for subsequent viewing (Advanced Workstation ADW 4.3, GE Healthcare Bio-Sciences, NJ, USA).

### Image analysis

PET/CT images were reviewed by consensus (PLK & HY) using an image fusion system (Advanced Workstation ADW 4.3, GE Healthcare Bio-Sciences, NJ, USA). Standard uptake value (SUV) was normalized by lean body mass for all patients. A fixed threshold set at SUVmax = 2.5 was adopted for the semi-automatic segmentation of primary tumors and nodal metastasis. This threshold was selected as it has been found to provide a reasonable correlation between metabolic tumor volume and morphologic volume, and well-accepted^11^. Necessary manual adjustment was made by deleting physiological or reactive uptakes in structures such as adjacent normal brain, normal salivary glands, Waldeyer’s lymphatic ring, etc so as to avoid over-segmentation (PLK & HY). Metabolic parameters, SUVmax and Metabolic Tumor Volume (MTV) of both primary tumors (T) and nodes (N) were recorded as SUVmax_T, MTV_T, SUVmax_N (of the hottest node) and MTV_N respectively.

MRI images of the primary cohort were reviewed by specialized head & neck radiologists (QYA & ADK), blinded to the PET/CT findings, by consensus on a workstation (Extended MR workspace, Philips medical system, Netherland B.V., The Netherland). Cervical nodes were identified based on morphologic criteria of 1) size, using short axis ≥5mm for retropharyngeal nodes, ≥11mm for jugulodiagastric nodes and 10mm for all other cervical nodes; 2) presence of necrosis or 3) extracapsular spread^12, 13^. Morphologic volume (VOL) of primary tumors (VOL_T) and all identified nodes (VOL_N) were calculated using summation-of-areas method by multiplying the slice thickness with cross-sectional area delineated from contrast enhanced T1-weighted images in axial planes.

### Radiological Staging

Patients were staged using American Joint Committee on Cancer (AJCC) TNM staging system (7^th^ edition). Primary tumor was assessed using MRI for tumor invasion for T-stage. Notably, patients with prevertebral space invasion were classified as T2 stage. For N-stages, all nodal metastasis identified using the above stated method were assessed with maximal axial length quantified in all 3 standard imaging planes. Specifically, supraclavicular regions for N3b were defined with reference to a guideline described before^14^. In addition, identification of any node with SUVmax ≥2.5 in PET images that did not reach morphologic criteria stated above was included for upstaging N-stages.

### Treatment protocol

All patients received standardized therapy protocol, i.e. Intensity Modulated Radiation Therapy (IMRT), with or without induction, concurrent and adjuvant chemotherapy, according to their clinical stages, and were followed up in accordance with oncologists specialized in the treatment of NPC (DLWK & VHFL).

#### Chemotherapy

Generally, for stage I/II disease, only IMRT was prescribed, while for stage III to IVB patients, concurrent chemotherapy (cisplatin 100 mg/m^2^ on day 1, 22 and 43) with or without adjuvant chemotherapy (cisplatin 80 mg/m^2^ on day 1 and 5-FU 1000 mg/m^2^ from day 1 to 4 for 3 cycles, starting 4 weeks after the completion of IMRT) were prescribed. For patients with bulky tumor, induction chemotherapy (cisplatin 100 mg/m^2^ on day 1 and 5-FU 1000 mg/m^2^ from day 1 to 5 for 3 weeks) was given with an aim to reduce tumor volume so that better balance could be achieved between radical dose delivered to tumors and unnecessary radiation exposure to organs-at-risk.

#### IMRT planning

For each patient, a thermoplastic cast and a customized mouth-guard were applied during above stated imaging and actual treatment. Planned dose was delivered using simultaneous accelerated radiation therapy technique (SMART) in 33~35 fractions (5 fractions per week, in consecutive weeks until designated dose is reached). Briefly, PET/CT images and MRI images were co-registered using a treatment planning system (Eclipse version 8.0 to 10.0 software, Eclipse Treatment Planning System, Palo Alto, CA.) for the delineation of targeted volumes including primary tumors and nodes, against organs-at-risk (OARs) including brainstem, spinal cord, globes, optic nerves, optic chiasm, lenses, temporomandibular joints, temporal lobes, auditory nerves, cochleae, mandible, oral cavity, larynx, parotid glands and vestibules. High risk regions (HRR) including the posterior half of the maxillary sinuses, nasal cavities, parapharyngeal spaces, styloid processes, basiocciput, basisphenoid, clivus, foramina rotunda and ovale, pterygopalatine fossae, pterygomaxillary fissures, infraorbital fissures, cavernous sinuses, and nodal stations (level Ib and level V) were also segmented. A total dose of 70 Gy was delivered to the targeted volumes enlarged by a 5mm margin, while a total dose of 66 Gy was prescribed to HRR with an enlarged margin of 3mm, considering inevitable motions, microscopic spread and set-up errors. Dose limits were set at 54 Gy for brainstem, optic nerves, and chiasm, and 45 Gy for spinal cord. On condition of some locally advanced diseases, dose limit for brainstem, optic nerves and chiasm could be escalated up to 60 Gy in order to maintain adequate dose delivery to targeted volumes. To parotid glands, mean dose was limited up to 26 Gy, while for the lenses and temporal lobes, minimalized dose was delivered on the premise that adequate dose was delivered to target organs.

### Clinical follow-up

Tissues blocks acquired by routine 6-site biopsies during nasoendoscopy (random biopsy at roofs, lateral and posterior walls of at bilateral nasopharynx) were microscopically investigated for response assessment of primary tumor. This assessment was performed at the 8th week after completion of IMRT, and repeated 2-weekly (maximum twice) upon the detection of malignant residue in the previous histological investigation. In cases of suspicious disease-persistence in cervical nodes, ultrasonography (USG) with fine-needle aspiration biopsy (FNAB) was performed. Disease persistence was diagnosed as detectable malignant residue in the 3^rd^ histological inspections (12 weeks after IMRT) or any USG guided FNAB, indicating an instant salvage treatment such as salvage surgery, intracavitary brachytherapy boost, stereotactic radiotherapy, etc. with an aim to reach complete response. Patients with no positive findings were diagnosed as complete local remission, and clinically followed every 3–6 month. Contrast-enhanced head & neck MRI and^18^F-FDG PET/CT scans were only indicated upon suspicious relapse or metastasis.

### Survival endpoints

Follow-up ended upon documentation of death or date of censor (Dec., 31, 2015). The primary survival endpoint was overall Survival (OS) which was calculated as a period between initial imaging diagnosis and documentation of death. Recurrence detected at nasopharynx, neck nodes and distant regions were deemed as local failure, regional failure and distant metastasis, respectively. Recurrence-free survival (RFS), as a secondary survival endpoint, was calculated as a period between initial imaging diagnosis and documentation of recurrence or death, whichever earlier. For patients with no reported endpoint events, their survival was censored upon the date of censor.

Additionally, to verify the positive findings that were found in the prospective cohort, an independent validation cohort of consecutive patients with newly diagnosed NPC (T_x_N_x_M_0_) who received contrast-enhanced Head & Neck MRI as pre-treatment evaluation within the same period and treated by standardized protocols from the same institution, were retrospectively reviewed. Conventional imaging modalities including diagnostic CT, MRI, etc. were used to exclude distant metastasis. MRI images for treatment planning of the validation cohort were reviewed by another team (VV & YH) using the same standard stated above, with volume of nodes and TNM stages recorded. The same survival endpoints were used for analysis, with the date of censor being Jan. 1, 2017.

### Statistical analysis

All statistical analysis was done by either SPSS (IBM SPSS Statistics, Version 23, IBM, New York, USA) or R (Version 3.2.3, The R Foundation for Statistical Computing, Vienna, Austria) with necessary analytical packages such as rpart, partykit, and survival installed directly from Comprehensive R Archive Network (CRAN).

Correlations of volume from MRI and PET were analyzed using Pearson’s correlation. Cox-regression model was adopted for the identification of independent prognostic factors with protocols stated as follows: Kaplan-Meier curves for categorical variables, i.e., gender, T-stage, N-stage, and the overall stage, were plotted and reviewed, with necessary combination upon similar survival curve results. Univariable followed by multivariable analysis were made to all parameters. Proportional hazards assumption was assessed by examining the Schoenfeld residuals. In the multivariable analysis, the presence of multicollinearity was assessed by a variance inflation factor (VIF). Factors suffering from multicollinearity were entered one at a time in the multivariable analysis. The factors that increased the predictive performances were also considered as independent predictive factors. Classification and Regression Tree (CART) analysis was conducted to derive a risk stratification rule with the hazard ratio of each risk-layer calculated using cox-regression model while statistical differences were compared with Kaplan-Meier model. Specifically, we entered only independent predictive factors in CART analysis, considering issues of the limited sample size and possibilities of over-fitting, and the tree were pruned with a complexity parameter (CP) at smallest x-error. The identified risk layers were cross-validated in an independent validation cohort using cox-regression model and Kaplan-Meier model. Benjamini-Hochberg procedure was adopted for multiple comparison correction. p < 0.05 was considered statistically significant.

### Data availability statement

The datasets analyzed during the current study available from the corresponding author on reasonable request.

## Results

### Patient demographics

A total of 200 patients (147 male, age 50 ± 13 years old, mean ± standard deviation, [S.D.]) were eligible for final analysis after excluding 25 patients from the initial recruitment presenting with confounding factors of synchronous malignancies (n = 1), distant metastasis (n = 14), technical data damage (n = 1), withdrawn from the designated IMRT or lost during follow-up (n = 11). For the validation cohort, 105 patients (74 male, age = 54.2 ± 14 years old, [mean ± S.D.]) were eligible for analysis. (see Table 1 for details of patient demographics).

**Table 1:** Patient’s demographics of both primary and validation cohorts.

Parameters	Primary Cohort	validation cohort
Gender		
Male	n = 147	n = 74
Female	n = 53	n = 31
Age/Years old (Mean ± S.D.)	50 ± 13	54 ± 14
Time between PET and MRI scans/days (Mean ± S.D.)	1.8 ± 1.0	N/A
Survival endpoint events		
Local recurrence	n = 12	n = 8
Regional recurrence	n = 6	n = 4
Distant recurrence	n = 26	n = 11
Death	n = 25	n = 13
Follow-up period/Months (Mean ± S.D.)	45 ± 22	49 ± 23
Treatment		
IMRT (70 Gy in 33~35 fractions)	n = 200	n = 105
Induction Chemotherapy	n = 73	n = 36
Concurrent Chemotherapy	n = 180	n = 83
Adjuvant Chemotherapy	n = 84	n = 45
Histological Types		
WHO Type 1	n = 1	n = 4
WHO Type 2	n = 5	n = 4
WHO Type 3	n = 194	n = 97
T-Stage (UICC/AJCC 7^th^ edition)		
T1	n = 61	n = 27
T2	n = 40	n = 15
T3	n = 68	n = 47
T4	n = 31	n = 16
N-Stage (UICC/AJCC 7^th^ edition)		
N0	n = 38	n = 18
N1	n = 84	n = 19
N2	n = 51	n = 54
N3	n = 27	n = 14
Overall-Stage (UICC/AJCC 7^th^ edition)		
I	n = 16	n = 7
II	n = 49	n = 12
III	n = 79	n = 57
IV	n = 56	n = 29

Abbreviations: S.D. = standard deviation, PET = positron emission tomography, MRI = magnetic resonance imaging, IMRT = Intensity-modulated radiation therapy.

### Patient-based correlation between metabolic and morphologic parameters

For the primary tumor, VOL_T was 17.9 ± 18.2cc (mean ± S.D.) while MTV_T was 19.4 ± 27.2cc. For cervical nodes, VOL_N was 16.5 ± 22.3cc whereas MTV_N was 12.2 ± 18.4cc. A strong linear correlation was detected between VOL_T and MTV_T (R = 0.873, p < 0.001) and VOL_N and MTV_N (R = 0.852, p < 0.001). (Additional data are given in Supplementary Table S1).

The correlations between SUVmax and volumetric parameters (MTV and VOL) were improved by a quadratic model compared to a linear model for both MTV_T (0.586 vs 0.690, R_linear_ vs R_quadratic_) and VOL_T (0.478 vs 0.545), and the same in cervical nodes for both MTV_N (0.696 vs 0.800) and VOL_N (0.540 vs 0.711).

### Survival analysis

Only 5 patients received salvage treatments due to disease persistence in the nasopharynx (n = 1) and cervical nodes (n = 4), and all subsequently achieved complete response. Five-year overall survival was 82% for the primary cohort, and 83% for the validation cohort.

No significant violation of proportional assumption was detected. Factors such as T-Stage (3.96, variance inflation factor), N-Stage (4.22), Overall Stage (5.15), MTV_T (5.47), MTV_N (5.63), VOL_T (5.54) and VOL_N (4.87) suffered from considerable multicollinearity.

Gender, age and all parameters derived from the primary tumor, i.e. T-stage, SUVmax, MTV_T and VOL_T, were found not predictive of OS or RFS. For OS, by univariable analysis, overall stage, MTV_N and VOL_N were predictive factors. Multivariable analysis found only VOL_N to be independently predictive of OS. For RFS, by univariable analysis, N-stage, the overall stage, VOL_N and MTV_N were predictive factors. Multivariable analysis found overall stage, N-stage and VOL_N to be independent predictive factors of RFS (Table 2).

**Table 2:** Survival Analysis.

Parameters	Univariable analysis		Multivariable analysis	
	HR(95% CI)	p	HR(95% CI)	p
**Overall survival as endpoint**				
Clinical Demographics				
Sex (male vs female)	0.65(0.28–1.50)	0.309	0.43(0.16–1.25)	0.085
Age	1.01(0.98–1.05)	0.397	1.02(0.99–1.05)	0.205
T-Stage	1.22(0.84–1.79)	0.294	1.22(0.63–2.37)	0.558
N-Stage	1.46(0.98–2.17)	0.064	0.68(0.30–1.53)	0.355
Overall Stage (III/IV vs I/II)	1.68(1.02–2.78)	0.042*	1.81(0.37–8.99)	0.466
Metabolic Parameters				
SUVmax (Primary Tumor)	1.01(0.93–1.10)	0.813	0.95(0.83–1.10)	0.494
MTV (Primary Tumor)	1.01(0.99–1.02)	0.280	1.02(0.98–1.06)	0.278
SUVmax (Cervical nodes)	1.07(0.99–1.16)	0.075	1.10(0.94–1.29)	0.259
MTV (Cervical nodes)	1.02(1.00–1.03)	0.048*	0.98(0.94–1.03)	0.452
Morphologic Parameters				
Volume (Primary Tumors)	1.01(0.99–1.03)	0.442	0.97(0.92–1.04)	0.406
Volume (Cervical nodes)	1.02(1.01–1.03)	0.002*	1.03(1.01–1.05)	0.014*
**Recurrence-free survival as endpoint**				
Clinical Demographics				
Sex (male vs female)	0.91(0.46–1.80)	0.782	0.60(0.28–1.30)	0.196
Age	1.00(0.97–1.02)	0.880	1.01(0.98–1.03)	0.657
T-Stage	1.18(0.89–1.57)	0.258	0.99(0.59–1.65)	0.956
N-Stage	1.39(1.02–1.90)	0.040*	0.49(0.26–0.91)	0.024*
Overall Stages (III/IV vs I/II)	2.92(1.23–6.93)	0.015*	4.22(1.23–14.53)	0.022*
Metabolic Parameters				
SUVmax (Primary Tumor)	1.01(0.95–1.08)	0.757	0.97(0.87–1.08)	0.534
MTV (Primary Tumor)	1.01(0.99–1.02)	0.352	1.02(0.99–1.06)	0.131
SUVmax (Cervical nodes)	1.07(1.01–1.14)	0.019*	1.08(0.96–1.20)	0.197
MTV (Cervical nodes)	1.02(1.01–1.03)	0.002*	0.99(0.96–1.02)	0.444
Morphologic Parameters				
Volume (Primary Tumors)	1.00(0.99–1.02)	0.669	0.97(0.92–1.01)	0.142
Volume (Cervical nodes)	1.02(1.01–1.03)	<0.001*	1.04(1.02–1.06)	<0.001*

*Denotes statistical significance.

Abbreviations: SUVmax = maximal standard uptake value, MTV = metabolic tumor volume, HR = hazard ratio, 95% CI = 95% confidence interval.

Due to a strong linear correlation between VOL_N and MTV_N, the parameters were found interchangeable, i.e. taking out VOL_N from the multivariable analysis resulted in MTV_N being independently predictive of RFS (HR = 1.02 [1.00–1.03, 95% CI], p = 0.026), and OS (HR = 1.02 [1.00–1.03], p = 0.048).

### Risk-stratification

**Figure 1:**
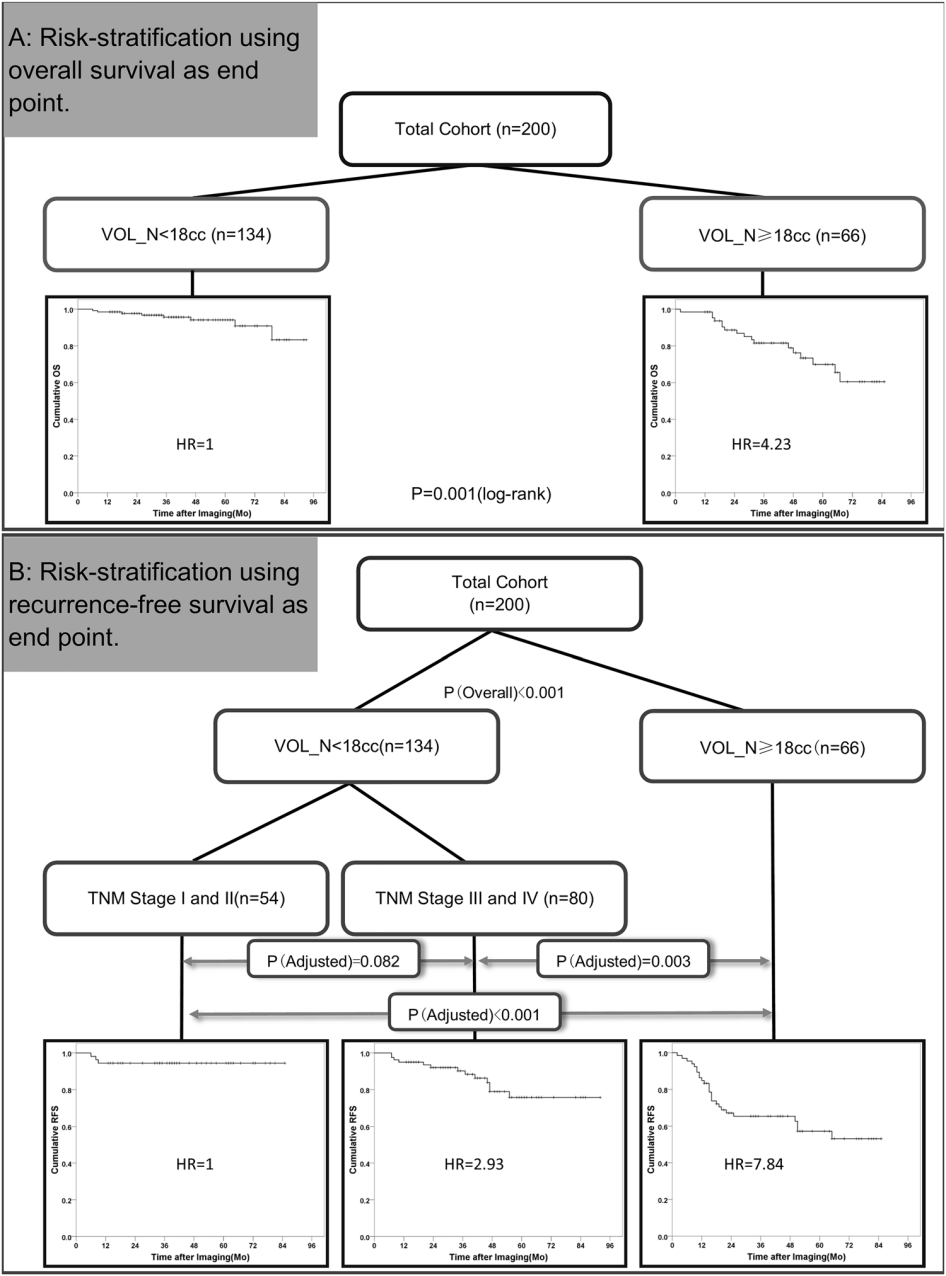
Risk stratification rules for patient survival.**(A)** Patient risk layers and the corresponding survival curves using OS as the endpoint. **(B)** Patient risk layers and the corresponding survival curves using RFS as the endpoint. Abbreviations: HR = hazard ratio, VOL_N = morphologic volume of cervical nodes, OS = overall survival, RFS = recurrence-free survival.

For OS, two risk-layers (Fig. 1A) were identified (p = 0.001): 1) VOL_N < 18cc (reference HR = 1), and 2) VOL_N ≥ 18cc (HR = 4.23). For RFS, three risk-layers (Fig. 1B) were identified (p < 0.001): 1) Overall Stage I/II with VOL_N < 18cc (reference HR = 1), 2) Overall Stage III/IV with VOL_N < 18cc (HR = 2.93), and 3) VOL_N ≥ 18cc regardless of disease stage (HR = 7.84). However, after multiple comparison correction, survival between the first 2 risk-layers (1 and 2 above) became not statistically significant (p = 0.082 after adjustment).

### Comparison of nodal volume based risk layers and TNM stage (7^th^ edition)

**Figure 2:**
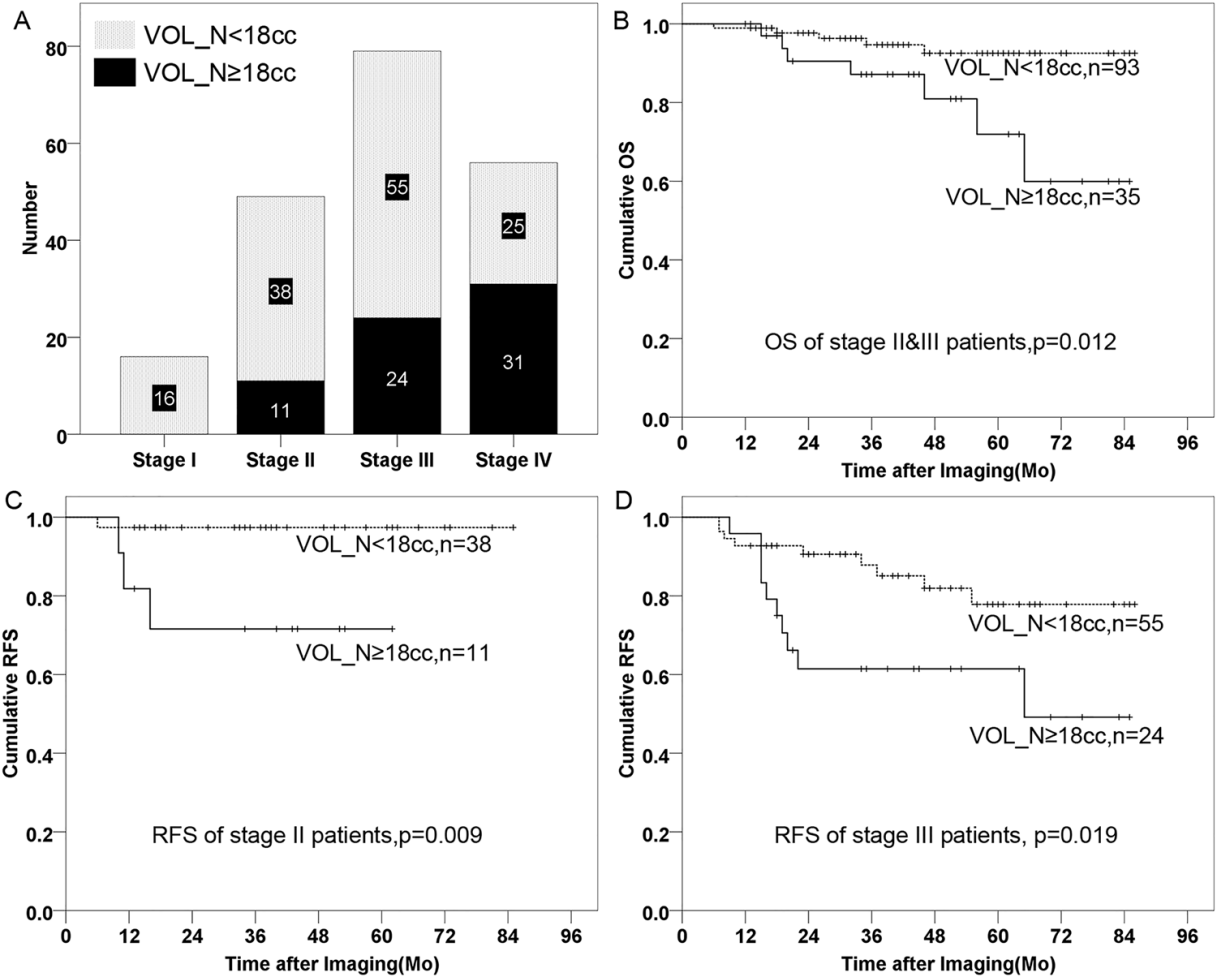
Distribution and survival of patients with the volume of cervical nodes ≥18cc and patients with the volume of cervical nodes < 18cc in the primary cohort.   (**A**) The distribution of patients with the volume of cervical nodes ≥18cc (black bar) and patients with the volume of cervical nodes<18cc (gray bar), stratified by the overall TNM stage (7th edition).  (**B**) Survival curves for stage II & III NPC patients with the volume of cervical nodes <18cc (dashed line) and the volume of cervical nodes≥18cc (solid line), using OS as the endpoint.  (**C**)  Survival curves for stage II NPC patients with the volume of cervical nodes <18cc (dashed line) and the volume of cervical nodes≥18cc (solid line), using RFS as the endpoint.  (**D**) Survival curves for stage III NPC patients with the volume of cervical nodes <18cc (dashed line) and the volume of cervical nodes≥18cc (solid line), using RFS as the endpoint. Abbreviations: VOL_N = morphologic volume of cervical nodes, OS = overall survival, RFS = recurrence-free survival. Mo = month.

66 patients in the primary cohort were identified with the highest risk level (VOL_N ≥ 18cc), using both OS and RFS as end-points, and these patients were staged as overall stage II (n = 11), stage III (n = 24) and stage IV (n = 31) using current TNM staging system (7^th^ edition) (Fig. 2A). Comparing to patients with VOL_N < 18cc in the same stage, patients were found to have significantly poorer survival in stage II and/or stage III diseases when VOL_N ≥ 18cc (HR = 3.91[1.24–12.33], p = 0.012 for stage II/III patients using OS as endpoint [Fig. 2B], HR = 11.05[1.15–106.44], p = 0.009 for Stage II patients using RFS as endpoint [Fig. 2C], and HR = 2.81[1.14–6.93], p = 0.019 for stage III patients using RFS as endpoint [Fig. 2D]). Hence, based on our threshold, 35 out of 128 patients (27.3%) in Stages II and III, and 17.5% of total cohort, would have a higher risk than suggested by the 7^th^ edition of the TNM staging system which uses 6 cm measurement for N staging (i.e. N1/N2 vs N3).

### Cross-validation of the identified threshold in the validation cohort

**Figure 3:**
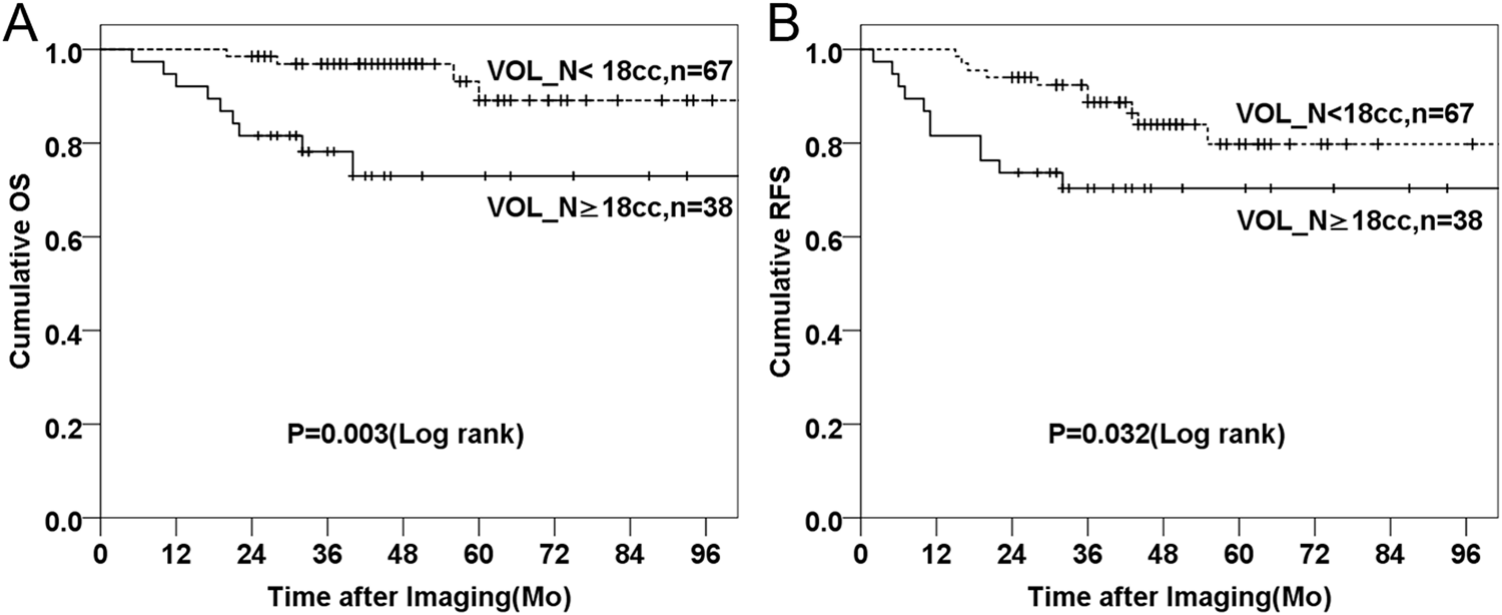
Risk layers in the validation cohort (n = 105). (**A**) Survival curves of patients with the volume of cervical nodes <18cc (dashed line) and the volume of cervical nodes≥18cc (solid line), using OS as the endpoint.  (**B**) Survival curves of patients with the volume of cervical nodes <18cc (dashed line) and the volume of cervical nodes≥18cc (solid line), using RFS as the endpoint. Abbreviations: VOL_N = morphologic volume of cervical nodes, OS = overall survival, RFS = recurrence-free survival. Mo = month.

Using the above identified threshold and risk-stratification rules, two risk-layers (Fig. 3A) were identified using OS as endpoint (p = 0.003): 1) VOL_N < 18cc (reference, HR = 1) and 2) VOL_N ≥ 18cc (HR = 5.09). Using RFS as endpoint, a modest trend (p = 0.062) was identified (Supplementary Fig. S1) using the above identified 3-risk layer: 1) stage I/II and VOL_N < 18CC, HR = 1; 2) stage III/IV and VOL_N < 18cc, HR = 2.87; and 3) VOL_N ≥ 18cc, regardless of TNM stages, HR = 6.03. However, considering the lack of significant survival differences between the first two layers (1 and 2 above), combining these two layers generated two risk-layers (Fig. 3B) with statistical significance: 1) VOL_N < 18cc (reference HR = 1) and 2) VOL_N ≥ 18cc (HR = 4.43, p = 0.032).

## Discussion

This study comprehensively evaluated quantitative parameters from staging MRI and PET/CT in a prospective cohort of non-metastatic NPC for prognostication and risk-stratification.

We found the cervical nodal volume to be the predominant risk factor of survival (both OS and RFS). These findings were validated in an independent cohort. On the other hand, all parameters related to the primary tumor were not predictive. Moreover, the nodal volume based on MRI was a stronger prognostic factor compared to metabolic tumour volume (MTV). Thus, in our cohort, we found no advantage of using PET/CT for this indication.

There are only a few studies that have evaluated the volume of cervical nodes in NPC, although nodal volume is well-accepted to be predictive in other head & neck cancers^15^. For NPC, Wang *et al*. found that patients with cervical nodes ≥10cc have a worse 5 year loco-regional control (85.0% vs 96.3%, p < 0.05)^16^. Recently, Luo *et al*. found that volume of cervical nodes was independently predictive of survival, albeit marginally in a cohort of T4Nx patients^17^. In our study, metabolic activity measured by SUVmax was found predictive only by univariate analysis but not independently predictive by multivariate analysis. Several prior reports, including from our own institution of a smaller cohort, have suggested that SUVmax of cervical nodes (and primary tumor) are prognostic using cut-off values of 6.5–7.58^,18, 19^. In this cohort, we have identified a stronger quadratic rather than a linear correlation between SUVmax and volume of cervical nodes, indicating a decreasing strength of correlation with SUVmax in larger nodes. We postulate that the presence of necrosis that was observed in the large lymph nodes confounded SUV values. Thus, the SUVmax of nodes may not be a reliable surrogate marker when nodal disease is large/advanced.

Based on the current TNM staging system, a dimension ≥6 cm upstages the N-stage to N3 and therefore Stage IVb. However, this measurement may not be applicable for imaging based assessment. Li *et al*. found only 1 out of 749 patients (0.13%) present with nodes reaching this size^10^. Moreover, matted/clustered nodes are not rare in advanced NPC cases, and whether the “6 cm standard” should be applicable to a single node or matted nodes remains ambiguous, resulting in inter-observer variability. Finally, although the axial length is expected to be a surrogate of volume, the unidimensional nature renders it only suitable for lesions with regular shapes^20^. Thus, volume measurements are highly suggested to be incorporated in the TNM staging system.

Our findings suggest three risk layers could be incorporated into TNM staging system such that N1 is defined as nodal volume smaller than 18cc with retropharyngeal nodes (regardless of laterality) and/or unilateral neck nodes, N2 as nodal volume smaller than 18cc with bilateral neck nodes, and N3 as nodal volume bigger than or equal to 18cc regardless of laterality. Using the threshold of morphologic nodal volume ≥18cc, 17.5% of patients would have been stratified to a higher risk than suggested by the current TNM staging system. Hence, the volume of 18cc may replace the “6 cm dimension” standard for N3 disease as stipulated by the current TNM system. A working-platform for automated or computer-assisted volume assessment by MRI would be critical in advancing the use of this important parameter as a quantitative measure, and image segmentation techniques which can be implemented in the clinics are on the horizon. On the other hand, metabolic volume of cervical nodes which was found to be highly correlated can be measured in a semi-automated way in the clinical PET workstation, and thus might be used as a surrogate of volume measurement for non-necrotic nodes.

A body of literature has found primary tumor parameters, volume and metabolic parameters such as SUV, MTV and total lesion glycolysis (TLG), to be prognostic, which is seemingly contradictory to our findings^6, 21, 22^. However, many of these studies did not include the analysis of nodal disease, and hence, their findings were not adjusted for nodal status. Primary tumor volume has been associated with regional and loco-regional failure-free survival based on a threshold of relatively high tumor volume, commonly 30–50cc, suggesting that the effect of primary tumor volume on survival is only evident when tumor volume exceeds this high cut-off ^6, 16^. For our cohort, it is notable that patients with primary tumor volume >48cc comprised only 5% of our cohort. Indeed, in a study of 1197 NPC patients, the impact of primary tumor bulk on OS was only evident when tumor volume was large^22^. In concordance with these findings, Chua *et al*. found tumor volume in early stage NPC to be not a significant predictor of survival^23^. It has been postulated that one of the factors would be the marked improvements in local control, resulting in the lessening impact of primary tumor features on survival especially when tumors were relatively small^22, 24, 25^. On the other hand, in locally advanced bulky disease, local control is more likely to be compromised due to inadequate dose delivery (<66.5 Gy) to part of the tumor bulk because of dose limitations arising from proximity with adjacent critical organs^25^. Hence, the failure of radiation delivery itself is the primary prognostic factor. Moreover, in our cohort, the comprehensive imaging work up, clinical follow-up and aggressive salvage treatment may have led to further improving local control, lessening the survival impact of the primary tumor per se.

We recognize the challenges and controversies in the methodology for segmentation of metabolic tumor volume, and that the selection of a fixed threshold for segmentation is arbitrary. We did not adopt adaptive threshold using the Fuzzy Locally Adaptive Bayesian (FLAB) based threshold, or percentage-based thresholds as there would be then no criteria for selection of reportable lesions. Moreover, percentage based thresholds are highly dependent on SUVmax, causing strong collinearity with volume measurement. Also, using percentage-based thresholds, metabolic volume is likely to be overestimated in low SUVmax lesions while underestimated in high SUVmax lesions, resulting in a weak correlation between metabolic volume and morphologic volume^11, 26, 27^. Hence, a fixed threshold was applied in our cohort. We did not incorporate Total Lesion Glycolysis (TLG) into survival analysis due to collinearity issues.

Our study is limited by the lack of a gold standard for determination of metastatic nodes by histological confirmation which is clinically not feasible. Instead, we have utilized standardized and well-accepted parameters by both MRI and PET/CT for the cohort, acknowledging the inherent limitations in false positive and false negative rates. Also, the validation cohort did not have PET/CT in addition to MRI scans. However, our aim was to verify the significant positive finding that nodal volume measured by MRI was an independent significant predictor for survival and that the 18cc volume threshold was robust in risk stratification.

In conclusion, morphologic nodal volume using MRI is an important factor in prognostication and risk stratification in NPC, and is suggested to be incorporated in the staging system. On the other hand, quantitative PET parameters have no role for this purpose in our cohort. However, in clinical practice, PET/CT has been found useful in detection of suspicious nodal involvement in small volume nodes for radiation treatment planning and for the exclusion of distant metastases in advanced disease.

## Electronic supplementary material

Supplementary Information
