# Supplementary material for: Cervical nodal volume for prognostication and risk stratification of patients with nasopharyngeal carcinoma, and implications on the TNM-staging system
Source: Sci Rep. 2017 Sep 4;7:10387. doi: 10.1038/s41598-017-10423-w (PMC5583337; doi:10.1038/s41598-017-10423-w)
Supplement: Supplementary file 1 — Supplementary Information [file 41598_2017_10423_MOESM1_ESM.pdf]

Title:

Cervical nodal volume for prognostication and risk stratification of patients with nasopharyngeal carcinoma, and implications on the TNM-staging system

Authors:

Hui Yuan, B.Med, M.Med[1]

Qi-Yong Ai, MBBS[2]

Dora Lai-Wan Kwong, MBBS, MD[3]

Daniel Yee-Tak Fong, PhD[4]

Ann D.King, MBBCh[2]

Varut Vardhanabhuti, MBBS[1.]

Victor Ho-Fun Lee, MBBS[3]

Pek-Lan Khong*, MBBS, MD[1]

1. Department of Diagnostic Radiology, Li Ka Shing Faculty of Medicine, The University of Hong Kong.
2. Department of Imaging & Interventional Radiology, Faculty of Medicine, The Chinese University of Hong Kong.
3. Department of Clinical Oncology, Li Ka Shing Faculty of Medicine, The University of Hong Kong.
4. School of Nursing, Li Ka Shing Faculty of Medicine, The University of Hong Kong.

Supplementary Table S1

Supplementary Table S1: Pearson's correlation between all quantitative imaging parameters

		T Stage	N Stage	Overall Stage	SUVmax_T	MTV_T	SUVmax_N	MTV_N	VOL_T	VOL_N
T Stage	R	1	0.084	**0.701**	**0.406**	**0.53**	0.030	<0.001	**0.634**	0.004
	P		0.235	**<0.001**	**<0.001**	**<0.001**	0.672	0.413	**<0.001**	0.951
N Stage	R	0.084	1	**0.608**	0.108	0.125	**0.674**	**0.659**	0.084	**0.705**
	P	0.235		**<0.001**	0.128	0.077	**<0.001**	**<0.001**	0.235	**<0.001**
Overall Stage	R	**0.701**	**0.608**	1	**0.333**	**0.414**	**0.398**	**0.382**	**0.483**	**0.414**
	P	**<0.001**	**<0.001**		**<0.001**	**<0.001**	**<0.001**	**<0.001**	**<0.001**	**<0.001**
SUVmax_T	R	**0.406**	0.108	**0.333**	1	**0.586**	**0.272**	0.095	**0.478**	0.021
	P	**<0.001**	0.128	**<0.001**		**<0.001**	**<0.001**	0.180	**<0.001**	0.771
MTV_T	R	**0.53**	0.125	**0.414**	**0.586**	1	**0.226**	0.106	**0.873**	0.078
	P	**<0.001**	0.077	**<0.001**	**<0.001**		**0.001**	0.135	**<0.001**	0.272
SUVmax_N	R	0.030	**0.674**	**0.398**	**0.272**	**0.226**	1	**0.696**	0.090	**0.540**
	P	0.672	**<0.001**	**<0.001**	**<0.001**	**0.001**		**<0.001**	0.203	**<0.001**
MTV_N	R	<0.001	**0.659**	**0.382**	0.095	0.106	**0.696**	1	0.048	**0.852**
	P	0.413	**<0.001**	**<0.001**	0.180	0.135	**<0.001**		0.502	**<0.001**
VOL_T	R	**0.634**	0.084	**0.483**	**0.478**	**0.873**	0.090	0.048	1	0.057
	P	**<0.001**	0.235	**<0.001**	**<0.001**	**<0.001**	0.203	0.502		0.424
VOL_N	R	0.004	**0.705**	**0.414**	0.021	0.078	**0.54**	**0.852**	0.057	1
	P	0.951	**<0.001**	**<0.001**	0.771	0.272	**<0.001**	**<0.001**	0.424	

Footnotes:
*Bold and underlined number denotes a statistical significance.
Abbreviations: SUVmax=maximal standard uptake value; SUVmax_T=SUVmax of primary tumor; SUVmax_N=SUVmax of cervical nodes; MTV_T= metabolic tumor volume of primary tumor, MTV_N= metabolic tumor volume of cervical nodes; VOL_T= morphologic volume of primary tumor; VOL_N= morphologic volume of cervical nodes.

Supplementary Figure S1

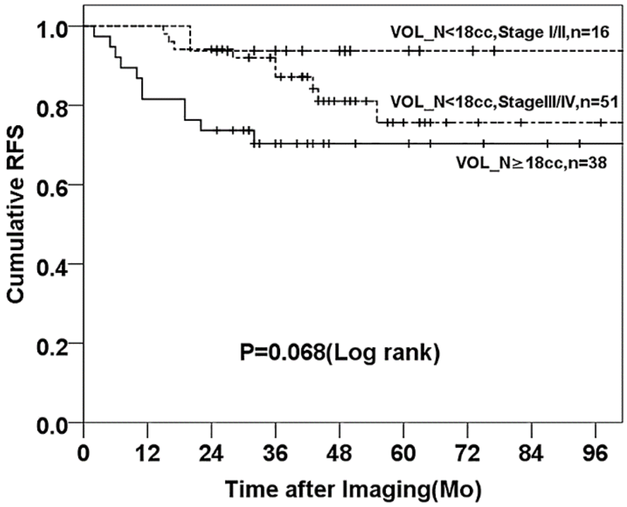

Figure Legends：

Supplementary Figure S1: Survival curves for patients using 3 risk-layers by further dividing patients with VOL_N<18cc into two groups (stage I/II and stage III/IV, TNM 7th edition) generate only moderate survival differences. Abbreviations: VOL_N= morphologic volume of cervical nodes, OS=overall survival, RFS=recurrence-free survival. Mo=month.
